# Diagnosis and treatment of incarcerated inguinal hernia with "complete reduction": a case report

**DOI:** 10.3389/fsurg.2026.1900000

**Published:** 2026-08-20

**Authors:** Haron Or Rashid Palash, Lide Tao, Muhammad Asad Iqbal, Anlai Ji

**Affiliations:** 1 Department of General Surgery, Affiliated Hospital of Yangzhou University, Yangzhou University, Yangzhou, Jiangsu, China; 2 Women’s Hospital, Zhejiang University School of Medicine, Hangzhou, China

**Keywords:** closed-loop obstruction, computed tomography, diagnostic laparoscopy, incarcerated inguinal hernia, reduction en masse, staged repair

## Abstract

**Background:**

Reduction en masse (REM) of an incarcerated inguinal hernia is a rare but serious diagnostic pitfall in which apparently successful reduction masks a persistent closed-loop obstruction within the preperitoneal space. Rather than returning to the peritoneal cavity, the hernia sac and its contents are displaced together as a single unit, so the constricting ring continues to compromise the trapped bowel. The result is an occult internal hernia that carries a high risk of bowel strangulation when diagnosis is delayed, yet the deceptively benign external examination frequently leads clinicians to attribute persistent symptoms to other causes.

**Case presentation:**

A 70-year-old man presented with small-bowel obstruction 48 hours after forcefully self-reducing a right inguinal hernia. The external bulge had resolved, but obstructive symptoms persisted and progressed. Contrast-enhanced computed tomography (CT) revealed a pathognomonic “preperitoneal hernia-sac sign": a rounded, obstructed jejunal loop adjacent to the inferior epigastric vessels. Diagnostic laparoscopy confirmed REM, with 10 cm of viable but oedematous small bowel trapped within a direct hernia defect. The constriction was released, a serosal tear was oversewn, and the internal ring was closed. Following conservative management of a postoperative pelvic haematoma, a second-stage open tissue repair was performed one week later. The patient recovered well and remained recurrence free at six-month follow-up.

**Conclusion:**

REM should be suspected in any patient with persistent obstructive symptoms after apparent hernia reduction, particularly when there is a history of forceful self-reduction. High-resolution contrast-enhanced CT demonstrating the “preperitoneal hernia-sac sign” is crucial for early diagnosis. Prompt laparoscopic exploration is both diagnostic and therapeutic, enabling assessment of bowel viability, release of the incarceration, and definitive repair while reducing postoperative morbidity. A staged repair strategy is safe and effective in contaminated or oedematous surgical fields. Greater clinician awareness and the systematic consideration of REM in emergency settings can prevent diagnostic delay and its serious consequences.

## Introduction

Reduction en masse is a rare condition in which an inguinal hernia is reduced together with its sac into the preperitoneal space, mimicking successful reduction (1). Self-reduction or repeated manual reduction is the commonest predisposing factor. Transient symptomatic relief creates a false sense of resolution and delays diagnosis, as the obstructed bowel remains unrecognized (2). Because incarceration persists, accurate preoperative diagnosis and prompt surgery are essential to prevent complications (3, 4). We report a 70-year-old man who developed small-bowel obstruction after forceful self-reduction of a right inguinal hernia, in whom diagnosis was delayed for 10 days despite characteristic CT findings.

## Case description

**Figure 1:**
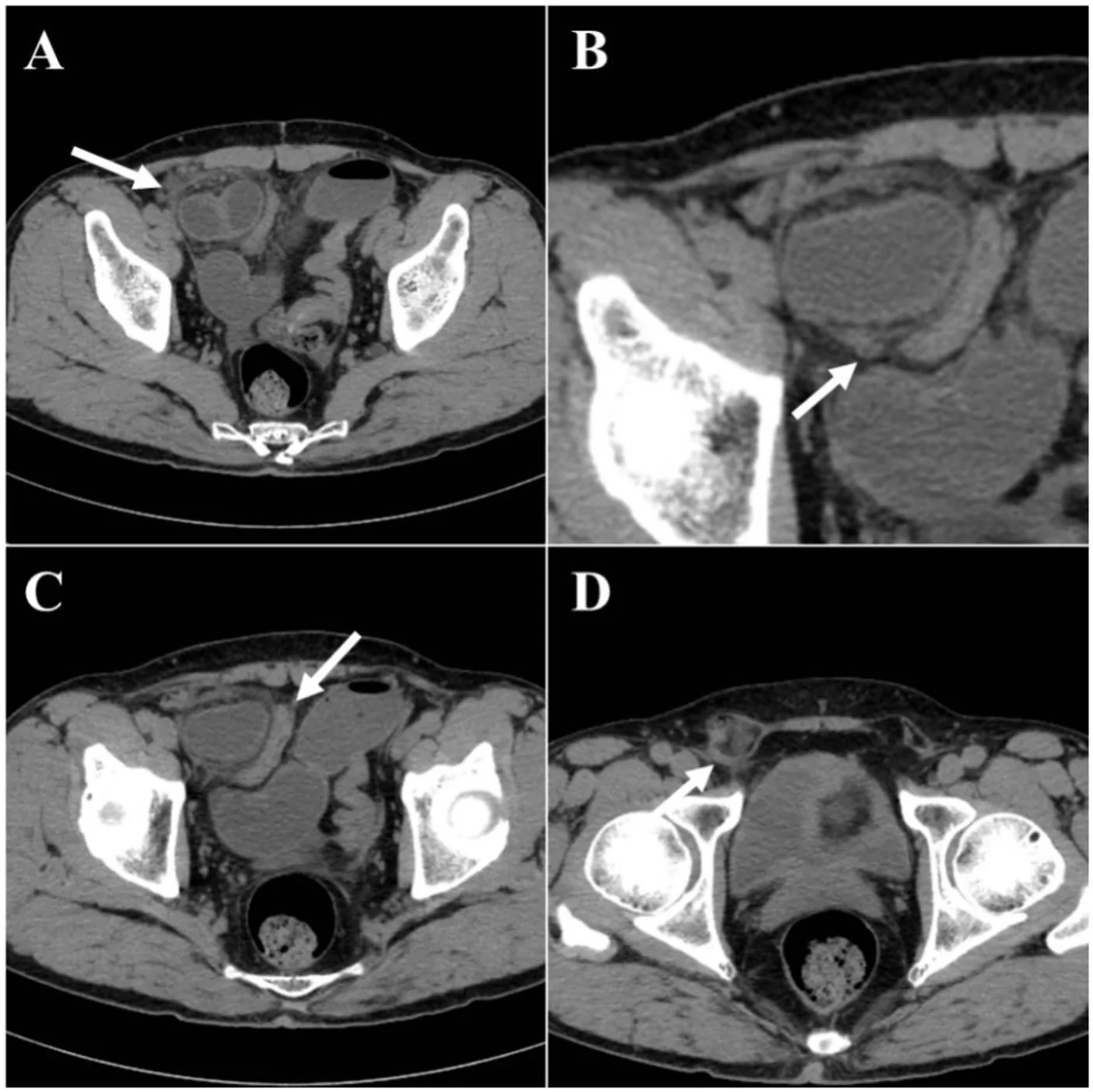
Initial abdominal CT scan images after the patient's onset. **(A)** The arrow indicates a spherical intestinal loop adjacent to the right groin. **(B)** The arrow indicates a circular cord-like structure at the site of obstruction. **(C)** The arrow indicates the bladder with a beak-like deformation along the closed intestinal loop. **(D)** The arrow indicates that the soft tissue shadow in the right groin area is more prominent than that on the left side.

**Figure 2:**
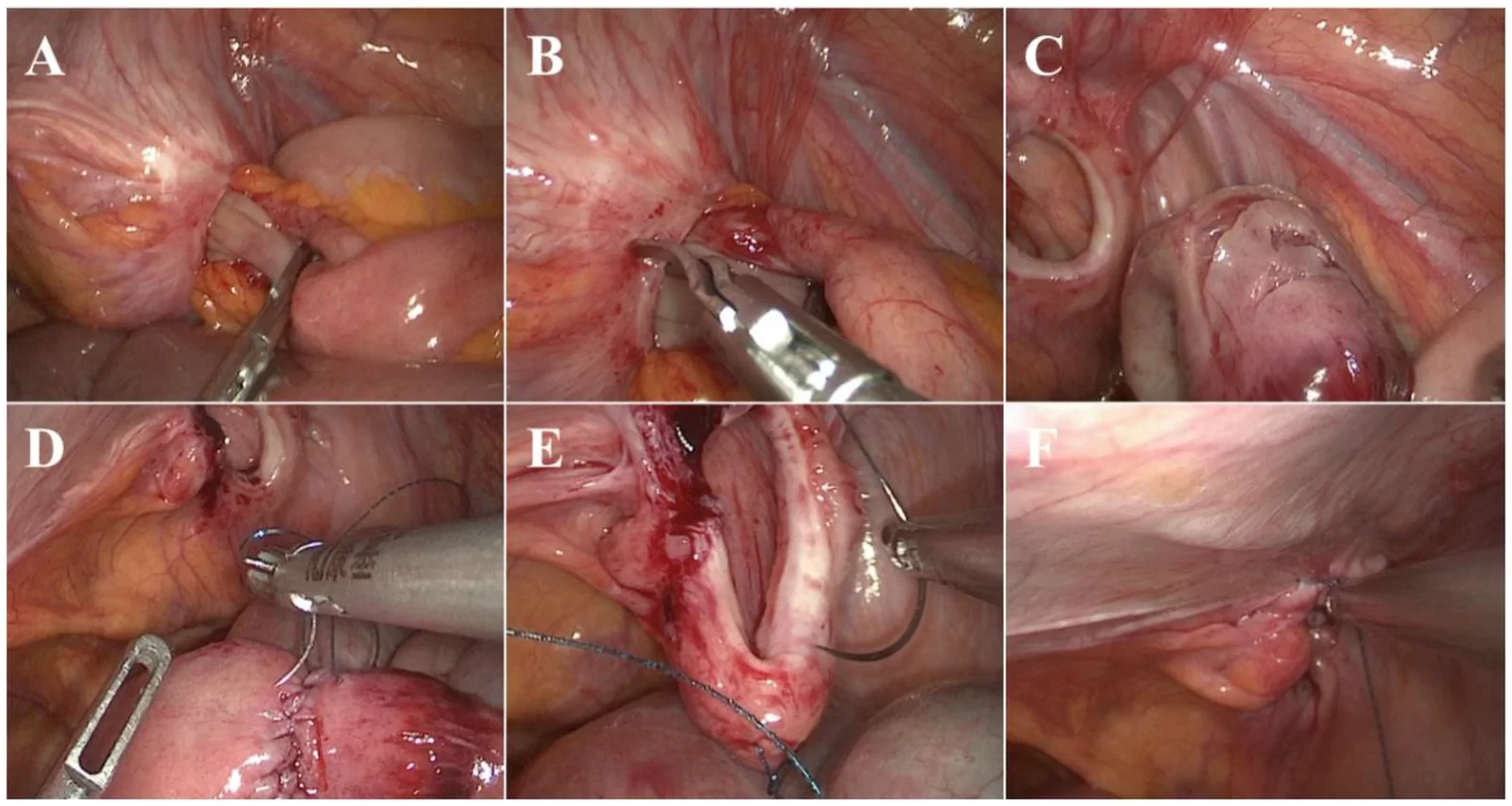
Intraoperative images from the patient's laparoscopic exploration. **(A)** A segment of the small intestine has herniated and is incarcerated. **(B)** Incision of the peritoneum at the hernial ring. **(C)** Damage to the serosa of the incarcerated bowel segment during retraction. **(D)** Repair of the damaged bowel serosa. **(E)** The distal peritoneum of the hernial sac is pulled back and sutured together with the peritoneum at the neck of the sac. **(F)** The hernial sac cavity has been closed.

A 70-year-old male was admitted complaining of lower abdominal pain and discomfort persisting for 5 hours. Clinical presentation included paroxysmal lower abdominal colic accompanied by nausea, vomiting, and cessation of both flatus and bowel movements. His medical history was significant for hypertension, managed with oral amlodipine besylate (with good control), and he had no history of other notable medication use. He had previously undergone transurethral resection of the prostate (TURP) and was diagnosed with a right inguinal hernia one year prior. The hernia had experienced multiple episodes of incarceration—most recently two days before the current onset—all of which were manually reduced by the patient himself without further specific treatment. Physical examination upon admission revealed: body temperature 36.7°C, pulse 80 beats/min, respiration 18 breaths/min, and blood pressure 140/80 mmHg. The patient was conscious and appeared to be in distress, with no jaundice of the skin or mucous membranes. Heart rhythm was regular, and breath sounds were clear. The abdomen was slightly distended; no visible bowel loops or peristaltic waves were observed. Tenderness was noted in the lower abdomen, but there was no rebound tenderness or muscle guarding; no abnormal masses were palpable, and shifting dullness was negative. Bowel sounds were active. No abnormal masses or significant tenderness were detected in the bilateral inguinal regions. Abdominal CT imaging showed narrowing of the small bowel lumen in the right iliac fossa, blurring of the surrounding mesenteric fat planes, and proximal small bowel dilation with fluid accumulation (Figure 1). Complete blood count results were: white blood cell count 11.88 × 10⁹/L, neutrophil percentage 88.2%, hemoglobin 169 g/L, and hematocrit 47.5%. The admission diagnosis was intestinal obstruction. Treatment included fasting, gastrointestinal decompression, anti-infection therapy, and fluid resuscitation. Two days later, the patient's abdominal pain subsided; he passed flatus and had two loose yellow stools. Conservative treatment was continued until the 10th day, but the intestinal obstruction failed to resolve completely. A follow-up CT scan showed worsening small bowel dilation and fluid accumulation compared to the initial scan. After consultation with the patient and his family, a decision was made to perform a laparoscopic exploratory laparotomy. Upon entering the abdominal cavity, a small amount of pale yellow fluid was observed in the pelvis, along with small bowel distension. A segment of the small intestine was found herniated into the right inguinal region; the bowel loop was entrapped by the neck of the hernial sac—formed by a ring of thickened peritoneum—located medial to the inferior epigastric artery. No intestinal adhesions or internal hernias were found. Based on the intraoperative findings and preoperative clinical presentation, the diagnosis was confirmed as a “right-sided incarcerated direct inguinal hernia with en bloc reduction” (Figure 2). The constriction was released, and the herniated bowel was retracted; the segment measured approximately 10 cm in length and exhibited wall edema. Although the serosa was damaged during retraction, peristalsis remained active, and there was no evidence of necrosis or perforation. After assessing that bowel resection was not indicated, the serosal tear was repaired, the distal hernial sac peritoneum was pulled back and sutured to the peritoneum at the sac neck, and the hernial sac cavity was closed. The patient passed flatus on the second postoperative day and successfully resumed an oral diet. One week later, the patient returned to the operating room for a Lichtenstein hernia repair under epidural anesthesia; the procedure proceeded without complications. The patient recovered well postoperatively and was discharged after suture removal one week later.

## Discussion

Incarcerated inguinal hernia is not uncommon in clinical practice; once diagnosed, emergency surgical treatment should be performed depending on the patient's condition (3). However, for elderly or frail patients—or those with severe underlying medical conditions that render emergency surgery extremely high-risk—manual reduction remains a vital therapeutic measure when the hernial contents are not necrotic. During the procedure, the application of pressure pushes the hernial contents back into the abdominal cavity, thereby relieving the incarceration; yet, a rare complication may occur: the hernial sac is displaced along with the contents away from the inguinal region—a phenomenon known as “en masse reduction.” A review of the literature indicates that this condition is most frequently observed in patients with chronic, reducible inguinal hernias, as its occurrence typically requires three conditions: a tough hernial sac neck, lax tissues surrounding the hernial ring, and a potential or pre-existing preperitoneal space (1). Due to long-term friction, the peritoneum at the neck of the hernia sac undergoes fibrosis and thickening driven by chronic inflammation, while the tissues surrounding the hernial ring become lax as a result of repeated herniation. Ultimately, under the pressure applied during reduction, the hernia contents and the sac pass through the lax hernial ring into a space within the preperitoneal area capable of accommodating the hernial mass. The patient in this case had a one-year history involving multiple episodes of incarceration, placing them at high risk for “en bloc” reduction during manual manipulation.

After complete reduction, the mass in the groin area appeared to disappear, but in fact, the incarceration was not relieved. Theoretically, the patient's clinical symptoms should not be relieved after reduction, but in fact, many patients in the literature, including this patient, experienced relief of discomfort symptoms such as pain, which masked the underlying condition. It is speculated that this may be related to the reduced pressure on the hernia sac after entering the preperitoneal space, resulting in a decrease in pressure within the hernia sac, or that the compression of the hernia contents was not severe during complete reduction, and the intestines remained patent. Subsequently, factors such as coughing and tension caused a sudden increase in intra-abdominal pressure, leading to more intestines entering the hernia sac and aggravating the incarceration (4), This may explain why the patient in this case did not exhibit symptoms of intestinal obstruction until two days after reduction. Although the mass in the inguinal region had disappeared, careful physical examination might still reveal a palpable mass upon deep palpation of the ipsilateral lower abdomen in some patients. As the condition progressed to a strangulated hernia, the patient developed symptoms and signs of peritonitis.

To reduce the risk of complications such as infection and recurrence, emergency surgery is often postponed after successful manual reduction of incarcerated inguinal hernias. Conservative treatment is implemented, and the patient is observed for complications such as intestinal necrosis and perforation. If no such complications occur, elective surgery is scheduled after the local inflammation has subsided and the patient is adequately prepared. However, complete reduction may delay the patient's condition, potentially leading to the development of a strangulated hernia. Therefore, early identification and complete reduction of incarcerated inguinal hernias are crucial. Imaging examinations are helpful for early diagnosis, especially CT scans, which have significant diagnostic value. However, due to insufficient understanding, the possibility of missed or misdiagnosis remains high. In this case, it was initially mistakenly believed that adhesions of part of the small intestine near the hernial ring due to chronic inflammation had caused adhesive intestinal obstruction. Furthermore, the patient consistently lacked signs of peritonitis such as abdominal muscle tension and rebound tenderness, suggesting no intestinal necrosis. Therefore, an initial conservative treatment plan was developed to wait for the obstruction to resolve spontaneously. Two days later, the patient's intestinal obstruction partially subsided, further reinforcing the clinical suspicion. Therefore, conservative treatment continued until the 10th day. After more than a week of conservative treatment, the patient's condition failed to fully improve, at which point the treatment plan was adjusted. This is one of the aspects requiring reflection during the diagnosis and treatment of this case. The CT imaging characteristics of en bloc reduction of incarcerated inguinal hernia can be summarized as the “preperitoneal hernia sac sign": 1. Closed-loop intestinal obstruction containing a bulbous intestinal loop; 2. Location adjacent to the groin area; 3. A round, cord-like structure at the site of obstruction, possibly thickened peritoneum at the hernial ring; 4. The bladder's beak-like shape along the closed intestinal loop, possibly due to deformation caused by compression between the hernia sac and peritoneum; 5. Prominent soft tissue in the groin area on the affected side, possibly formed by the remaining portion of the hernia sac or swelling of the soft tissue in the groin area (4, 5). A retrospective review of the initial abdominal CT images obtained after the onset of the disease revealed the characteristic imaging features (Figure 1); however, due to a lack of familiarity with the condition, the radiologist at the time failed to provide a precise diagnosis, and the clinicians also failed to recognize it promptly.

As the condition progresses, if clinical symptoms fail to resolve completely—or even worsen—following reduction, surgery becomes the definitive treatment. The specific surgical approach should be determined based on a comprehensive assessment of the patient's condition and available medical capabilities; intraoperative exploration revealing incarcerated hernia contents within the sac makes the diagnosis straightforward. Care must be taken to avoid oversight; there have been reports of anterior approach surgeries for incarcerated composite inguinal hernias where only a direct hernia was identified and repaired, while an indirect hernia—which had been reduced en bloc—was overlooked, ultimately leading to intestinal necrosis and perforation, with the correct diagnosis only established during a subsequent operation (6). While an inguinal incision facilitates hernia repair, the limited surgical field makes thorough exploration difficult, potentially leading to missed diagnoses; therefore, a standard exploratory incision is recommended. Laparoscopic exploration offers the advantages of minimal invasiveness and a wide field of view, allowing for a comprehensive assessment of the abdominal cavity and enabling immediate therapeutic intervention—such as laparoscopic transabdominal preperitoneal (TAPP) hernia repair—once the diagnosis is confirmed. Even in cases where conversion to open surgery becomes necessary due to technical constraints or clinical requirements, the laparoscopic approach aids in planning the incision to minimize trauma, and it is increasingly utilized in cases where the preoperative diagnosis is unclear (1, 7, 8). Once the diagnosis was confirmed, the incarcerated contents were released as soon as possible, and appropriate management was determined based on the viability of the herniated contents and the degree of contamination in the inguinal region. In this case, the incarcerated bowel wall exhibited significant edema, and the serosa was damaged during retraction; given the potential for bacterial translocation and contamination in the inguinal area, the placement of a mesh carried a risk of uncontrollable infection. Consequently, a staged surgical approach was adopted. To prevent recurrent incarceration prior to the second-stage procedure, the distal peritoneum of the hernial sac was retracted and sutured together with the peritoneum at the sac neck during the initial laparoscopic operation, thereby closing the hernial sac cavity. The second operation was performed one week after the first; at that time, performing TAPP or TEP would have been technically challenging and associated with a high risk of complications due to persistent local peritoneal edema, so an open Lichtenstein hernia repair was selected for the second stage.

## Conclusion

Due to the deceptive nature of the clinical presentation, cases of en bloc reduction of incarcerated inguinal hernia—including the present case—are frequently not diagnosed in a timely manner in the literature. Early recognition and prompt surgical intervention can improve patient outcomes and reduce medical costs; therefore, clinicians should heighten their awareness of this condition. Clinicians should consider this diagnosis when symptoms fail to resolve completely following manual reduction of an incarcerated inguinal hernia. Any suspicious clinical sign—such as a palpable mass felt above the inguinal region after reduction—should not be overlooked; instead, a careful physical examination should be performed for confirmation, accompanied by timely imaging studies such as abdominal CT. Clinicians should personally review the imaging scans rather than relying solely on the radiologist's report for diagnosis and management. With a thorough understanding of the condition, careful analysis of clinical manifestations, and the use of imaging modalities like CT, establishing a definitive diagnosis is not difficult. When the diagnosis remains uncertain or the clinical course necessitates surgical intervention, the surgical approach should be tailored to the specific circumstances; laparoscopic exploration is the preferred option when conditions permit, offering the advantages of minimal invasiveness, a wide field of view, and the flexibility to adjust the surgical plan intraoperatively.

## Data Availability

The original contributions presented in the study are included in the article/Supplementary Material, further inquiries can be directed to the corresponding author.
