# Correction: Diagnosis and treatment of incarcerated inguinal hernia with “complete reduction": a case report

**DOI:** 10.3389/fsurg.2026.1981170

**Published:** 2026-09-09

**Authors:** Frontiers Production Office

**Affiliations:** Frontiers Media SA, Lausanne, Switzerland

**Keywords:** closed-loop obstruction, computed tomography, diagnostic laparoscopy, incarcerated inguinal hernia, reduction en masse, staged repair

Affiliation of reviewer Vinicio Rizza was incorrectly written as “Azienda Usl Teramo, Italy”. The correct affiliation is “ASL 1 Avezzano Sulmona L'Aquila, Italy”.

The original version of this article has been updated.

